# A case of invasive micropapillary carcinoma of the breast involving extensive lymph node metastasis

**DOI:** 10.1186/1477-7819-12-84

**Published:** 2014-04-04

**Authors:** Kenji Taketani, Eriko Tokunaga, Nami Yamashita, Kimihiro Tanaka, Yoko Zaitsu, Sayuri Akiyoshi, Satoko Okada, Shinichi Aishima, Masaru Morita, Yoshihiko Maehara

**Affiliations:** 1 Department of Surgery and Sciences, Graduate School of Medical Sciences, Kyushu University, 3-1-1, Maidashi, Higashiku, Fukuoka 812-8582, Japan

**Keywords:** Invasive micropapillary carcinoma, Prognostic factor

## Abstract

We herein report a case of invasive micropapillary carcinoma (IMPC) involving extensive lymph node metastasis with no recurrence for over 7 years. A 41-year-old female presented with pain and a swelling mass in the left axillary region, which had been present for several months. The tumor measured 1.6 cm in diameter in the middle of upper area of the left breast. Based on the findings of a core needle biopsy the pathological diagnosis was IMPC or mucinous carcinoma. The cytology of the left axillary lymph node was positive for metastatic carcinoma. The patient underwent a left mastectomy and a left axillary dissection (level I to III). The postoperative pathological diagnosis was IMPC with mucin production, and the number of metastatic lymph nodes was 59. The patient was given adjuvant chemotherapy (four courses of 5-fluorouracil, epirubicin and cyclophosphamide (FEC) and four courses of docetaxel), radiation for the left chest wall, supraclavicular and internal thoracic area, and then received tamoxifen for 5 years. The patient has remained recurrence-free for over 7 years. IMPC is known to be an aggressive histological type associated with a high incidence of lymph node metastasis and a poor prognosis. It seems that long-term survival was obtained by performing sufficient medical treatment. Prognostic factors other than the number of lymph node metastases may also exist.

## Background

Invasive micropapillary carcinoma (IMPC) was described by Fisher as an invasive papillary cancer with an exfoliative appearance [1]. Pettinato *et al*. reported an invasive ductal carcinoma resembling ovarian serous carcinoma as a ‘pseudopapillary carcinoma of the breast’ [2]. IMPC was first reported by Siriaunkgul and Tavassoli in 1993 as a rare subtype of epithelial tumor of the breast [3]. The percentage of IMPC in all breast cancers is estimated to be 1.7% to 2.7% [4,5]. IMPC is characterized by an inside-out growth pattern forming a micropapillary or tubular-alveolar arrangement [3], invasion to the lymphatic or vascular space and a high frequency of lymph node metastasis [4,6-8].

IMPC is associated with a poor prognosis. Luna-More *et al*. reported that of 27 patients with micropapillary carcinoma, all of them had metastasis to axillary lymph nodes. Twelve of these patients were followed-up, six of whom died after an average of 22 months from the initial treatment [4].

We herein report a patient with IMPC who had 59 lymph node metastases, and who has remained free of recurrence for over 7 years.

### Case presentation

The present case was a 41-year-old premenopausal female at presentation. The patient had experienced pain and a swelling mass in the left axilla for several months before visiting a local doctor. A nodule was detected in the middle of upper area of the left breast, and a core needle biopsy was performed, which indicated a pathological diagnosis of IMPC or mucinous carcinoma. The cytology of the left axillary lymph nodes was positive for metastatic carcinoma. The patient was referred to Kyushu University Hospital, Fukuoka, Japan, for the purpose of additional examinations.

**Figure 1:**
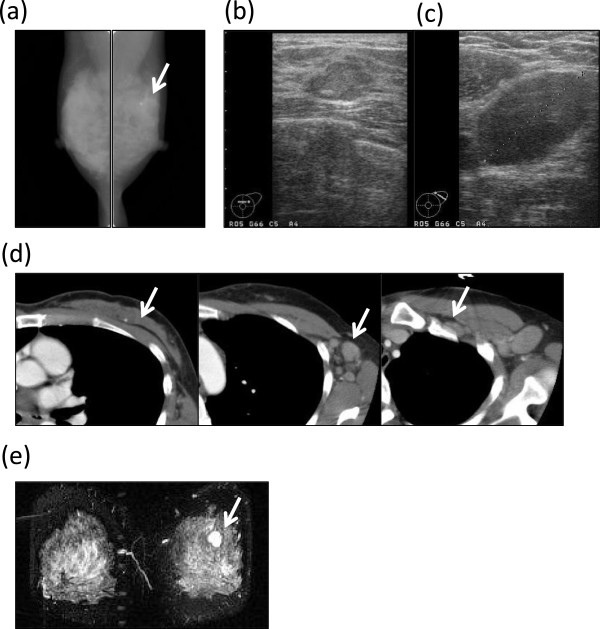
**Imaging findings. (a)** Mammography showed focal asymmetrical density in the left upper area of the left mediolateral oblique position (arrow). **(b)** Ultrasonography confirmed the presence of a 21 mm in diameter, lobulated, low- to iso-echoic area with a high echo spot, the border of which was unclear. **(c)** The ipsilateral axillary lymph nodes were swollen, the largest of which was 32 mm in diameter. **(d)** A CT scan revealed a low enhanced mass with calcification in the middle of upper area of the left breast (left), and the ipsilateral axillary (middle) and subclavicular lymph nodes (right) were swollen. **(e)** MRI revealed a lobulated mass with high intensity in the middle of upper area of the left breast.; CT, computed tomography; MRI, magnetic resonance imaging.

A physical examination revealed that a 2 cm in diameter hard mass was palpable in the AC area of the left breast, and painful, hard lymph nodes were palpable in the patient’s left axilla. The laboratory data showed a high level of carbohydrate antigen 15-3 (CA15-3; 39.8 U/mL), but normal levels of carcinoembryonic antigen (CEA; 2.0 ng/mL) and National Cancer Center-ST439 (NCC-ST439; 4.1 U/mL). Mammography showed focal asymmetrical density in the left upper area in the left mediolateral oblique position (Figure 1a). Ultrasonography confirmed a lobulated, low- to iso-echoic nodule with a high echo spot (Figure 1b). It was noted to be 21 mm in diameter and the border was unclear. The ipsilateral axillary lymph nodes were swollen, the largest of which was 32 mm in diameter (Figure 1c). A computed tomography (CT) scan revealed that the low enhanced mass with calcification was in the middle of upper area of the left breast (Figure 1d, left), and that the ipsilateral axillary (Figure 1d, middle) and subclavicular (Figure 1d, right) lymph nodes were swollen. There were no findings of metastasis to the bone, liver or lungs. Magnetic resonance imaging (MRI) revealed the presence of a lobulated mass with high intensity in the middle of upper area of the left breast, which was reinforced by gadolinium from the early phase (Figure 1e).

Based on these clinical and pathological findings, the patient was diagnosed to have an IMPC or mucinous carcinoma according to The Japanese Breast Cancer Society’s *General Rules for Clinical and Pathological Recording of Breast Cancer*[9] and the clinical stage was T1cN3aM0 Stage IIIC according to the Union for International Cancer Control (UICC) TNM Classification of Malignant Tumors.

**Figure 2:**
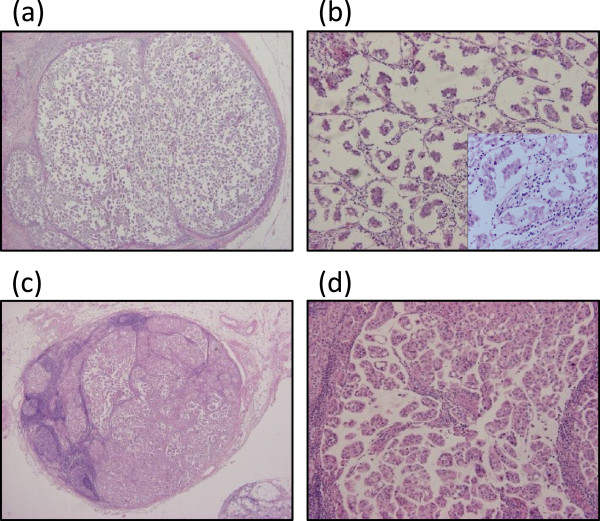
**Pathological findings of H & E staining. (a,b)** The primary lesion had a micropapillary component and clusters of carcinoma cells floating in extracellular mucin pools. The histological diagnosis was IMPC with mucin production. (a) × 40 and (b) × 100 and × 400 (in the square) magnification. **(c,d)** The metastatic lymph nodes showed similar pathological features as the primary lesion. (c) × 40 and (d) × 100 magnification. H & E, hematoxylin and eosin; IMPC, invasive micropapillary carcinoma.

The patient underwent a left mastectomy and a left axillary lymph node dissection (level I to III). A histopathological examination of the postoperative specimen showed that the masses of the micropapillary component and clusters of carcinoma cells were floating in extracellular mucin pools. The histological diagnosis was IMPC with mucin production (Figure 2). The histopathological findings showed that the histological nuclear grade was 1 (nuclear atypia 2 and mitotic count 1). Fifty-nine lymph nodes were diagnosed to be positive for carcinoma cells: 41 in level I, 12 in level II and six in level III. Lymph duct and vessel invasion were not observed.

**Figure 3:**
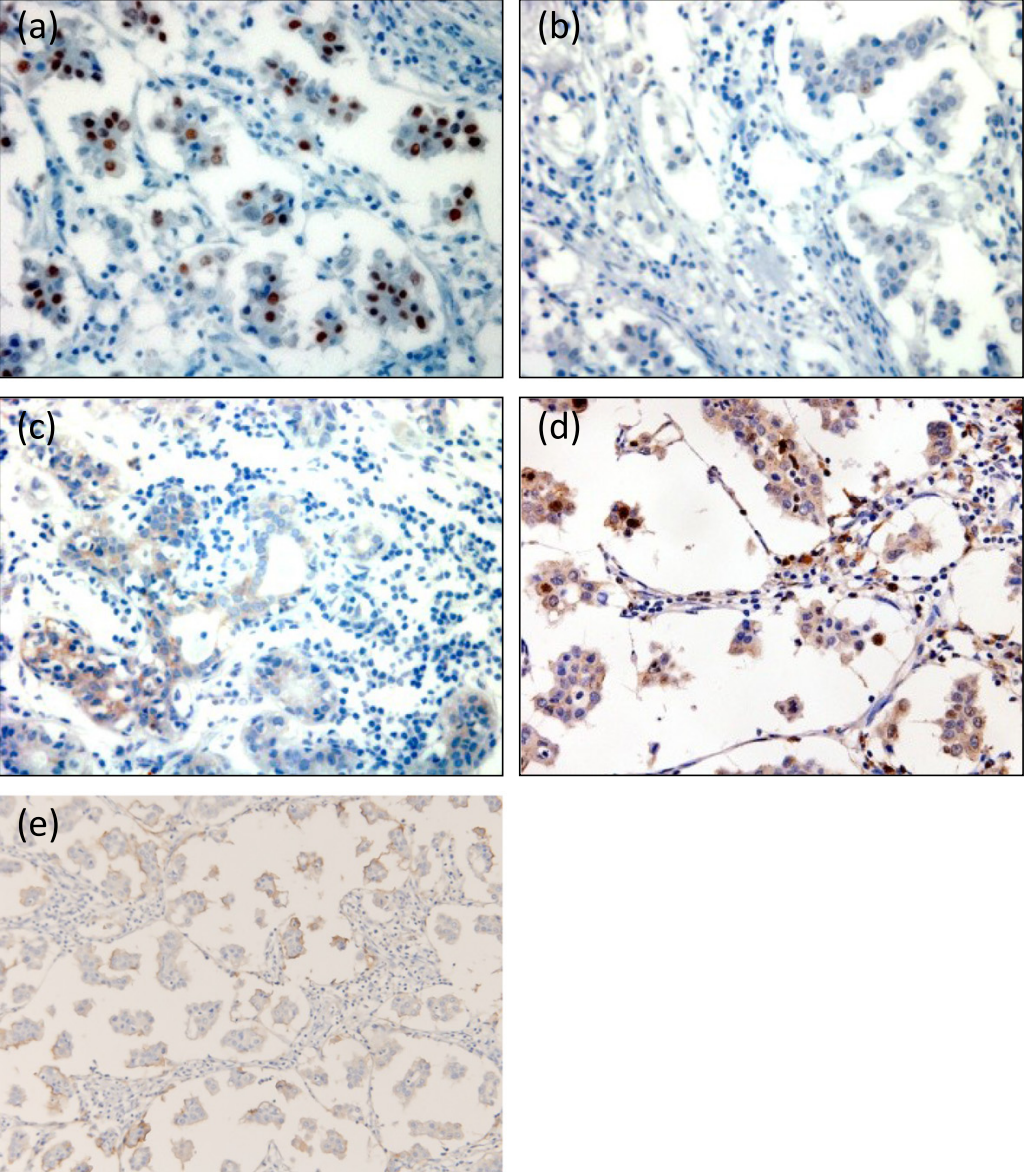
**Immunohistochemical findings of ER, PgR, HER2, Ki-67 and CD24. (a)** ER, **(b)** PgR, **(c)** HER2, **(d)** Ki-67 and **(e)** CD24. The cells were positive for the ER and for CD24, negative for the PgR and HER2, and the Ki-67 labeling index was 4.3%. × 400 magnification. ER, estrogen receptor; PgR, progesterone receptor.

The immunohistochemical examination of the tumor cells showed positivity for the estrogen receptor (ER; Allred score, proportion score (PS) 4 + intensity score (IS) 2 = total score (TS) 6), and negative findings for the progesterone receptor (PgR; Allred score, PS 0 + IS 0 = TS 0) and HER2 (score 0). The Ki-67 labeling index was 4.3% (Figure 3).

The patient was treated with four courses of 5-fluorouracil (5-FU), epirubicin and cyclophosphamide (FEC) (5-FU 500 mg/m^2^, epirubicin 100 mg/m^2^, cyclophosphamide 500 mg/m^2^) and four courses of docetaxel (75 mg/m^2^) as adjuvant chemotherapy. Radiation therapy to the left chest wall and left cervical lesion (30.6 Gy/17 Fr and 20 Gy/10 Fr) and the left internal thoracic lymph nodes (50 Gy/25 Fr) was added. Thereafter, the patient took tamoxifen for 5 years. The patient has remained free from relapse for over 7 years after the surgery.

## Discussion

We herein report a case of IMPC with no recurrence for over 7 years despite the presence of extensive lymph node metastasis. IMPC is a relatively new disease concept, and is a rare subtype of epithelial tumor of the breast. Most cases of IMPC are associated with nodal metastases and a poor prognosis [6].

Metastases to axillary lymph nodes were seen in 71.2% to 100% of IMPC cases [4,10,11]. The metastases were typically multiple, with 51% of cases having three or more positive lymph nodes [6], and the average number of metastatic lymph nodes was 10.7 [11]. In addition, lymphatic and vascular invasion has been reported in 33% to 67% of cases [3,4,8]. The number of nodal metastases is associated with the prognosis in breast cancer, regardless of the pathological type [12]. Nassar *et al*. reported that the nodal status and skin involvement were the only parameters that predicted a poor prognosis in IMPC [6]. The prognosis of IMPC is significantly worse than that of invasive ductal carcinoma of the scirrhous type, and the 5-year overall survival rate of IMPC is 50.5%, compared to 85.6% in scirrhous type cases [11]. The 5-year recurrence rate was 62.6% for IMPC and 24.0% for scirrhous type tumors [11]. However, a recent study showed that the disease-specific survival rate in IMPC was similar to that of invasive ductal carcinoma (91.9% versus 88%) [13]. In the report, IMPC with ER negative and lymph node metastasis (≥4) was significantly associated with worse 5-year disease-specific survival and 5-year overall survival [13].

The mechanism underlying the high incidence of lymph node metastasis in IMPC has not been fully elucidated. Ide *et al*. reported invasive carcinoma of the breast comprising an IMPC component with significantly higher incidence of lymph node metastasis and invasion [14]. The tumors involving an IMPC component, which is positive for hormone receptors and negative for HER2, tend to have a higher incidence of nodal metastasis compared with their counterparts in all invasive carcinomas. In contrast, lesions containing an IMPC component and classified as hormone receptor negative and HER2 positive have a lower incidence of nodal metastasis [14]. CD24 has been reported as another marker for IMPC related to lymph node metastasis. CD24 is an adhesion mucin-like molecule, and its expression could increase the ability of cancer cells to metastasize [15] and invade lymph nodes [16]. In our case, the expression of CD24 was positive in the epithelial lesion of the mammary duct (Figure 3e). Our case was positive for both ER and CD24, but negative for HER2, and the number of lymph nodes with metastasis was 59, agreeing well with the previous descriptions.

We had considered that the prognosis of this patient would be very poor. However, more than 7 years have passed since the patient’s surgery without relapse. We were therefore interested in determining whether there was any particular reason for the comparatively good prognosis of this patient.

We had performed a complete axillary node dissection from level I to III, and added standard chemotherapy (anthracycline and taxane), endocrine therapy and radiotherapy to the left chest wall and supraclavicular and internal thoracic regions in the present patient. Although there was no contribution of axillary lymph node dissection to the improvement of the survival of the breast cancer patients as determined by a meta-analysis [17], it does seem to be important for local control [18]. Radiation therapy followed by mastectomy has been previously reported to contribute to a better survival rate in patients with breast cancer who have more than four positive lymph nodes [19]. These factors may have been associated with the survival of the present patient. As for the endocrine therapy, the age of this patient was around 40 years old, and considering the number of axillary lymph node metastases, ovarian suppressive function using luteinizing hormone-releasing hormone (LH-RH) agonist might be suitable for this case. This patient was premenopausal before starting chemotherapy; however, the chemotherapy-induced amenorrhea occurred during chemotherapy. There were no data that show the superiority of the tamoxifen plus LH-RH agonist compared to tamoxifen alone after chemotherapy for females older than 40 years of age [20]. Therefore, we did not introduce the LH-RH agonist for this patient. Tamoxifen was given for 5 years as the standard method at that time, although the Adjuvant Tamoxifen: Longer Against Shorter (ATLAS) trial showed that the clinical outcome of 10 years’ intake was better than 5 years’ intake in recurrence and mortality, particularly after 10 years [21].

On the other hand, the intrinsic subtype is also considered to be an important prognostic factor [22,23]. The prognosis of the luminal A type breast cancer is excellent compared with HER2 positive and triple negative breast cancers. In IMPC, ER positivity has also been demonstrated to be related to a good prognosis [24]. In our case, the tumor was positive for the ER, and negative for the PgR and HER2. The Ki-67 labeling index was only 4.3%. From these IHC markers, this case seems to be classified into a ‘luminal A’ type breast cancer according to the St Gallen International Expert Consensus, 2011 [25]; although Prat *et al*. recently suggested that ‘luminal A’ in immunohistochemical-based definition is hormone receptor positive/HER2 negative/Ki-67 less than 14%, and PgR more than 20% [26]. Negativity of PgR may be related to the worse prognosis; however, a very low Ki-67 labeling index might be related to better prognosis. It is considered that adjuvant chemotherapy is unnecessary for luminal A type breast cancer without lymph metastasis [27]. However, even when the tumor is luminal A type, chemotherapy is considered necessary if the case is of high risk of recurrence [26]. This case was positive for extensive axillary lymph node metastasis, and the age of the patient was young. Therefore, we decided to add the standard adjuvant chemotherapy of anthracycline and taxane. Further study is required for the indication of the chemotherapy adding to the hormone therapy for the luminal type of breast cancer.

Zhao *et al*. reported that vascular endothelial growth factor (VEGF)-C promotes the proliferation of peritumoral lymphatic vessels, and that lymphatic invasion and lymph node metastasis are frequently induced in IMPC [28]. In lymphatic metastasis, the cancer cells in the primary site migrate in lymphatic vessels and then adhere to the endothelial lining, which leads to extravasation and the formation of secondary tumor sites. MUC-1, referred to as epithelial membrane antigen (EMA), is a heavily glycosylated transmembrane glycoprotein and expressed on the apical surface of a wide variety of epithelial cells. MUC-1 was reported to be very important in adhesion to endothelial lining [29]. The partial reverse cell polarity in breast carcinoma was reported to be associated with the decrease of immunostaining for MUC-1 and lymphatic tumor spread [30]. We did not examine the immunostaining of MUC-1; however, there may be a relationship between extensive lymph node metastasis and VEGF-C and MUC-1.

## Conclusions

At present, it can be concluded that the thorough surgical resection, completion of standard chemotherapy and hormone therapy, radiation to the chest wall and the regional lymph node areas, and ‘luminal A’ subtype might have contributed to the good prognosis of this patient; although other so-far unidentified prognostic factors may also exist.

## Consent

Written informed consent was obtained from the patient for publication of this Case report and any accompanying images.

## Abbreviations

5-FU: 5-Fluorouracil; ATLAS: Adjuvant Tamoxifen: Longer Against Shorter; CA15-3: Carbohydrate antigen 15-3; CEA: Carcinoembryonic antigen; CT: Computed tomography; EMA: Epithelial membrane antigen; ER: Estrogen receptor; FEC: 5-Fluorouracil epirubicin and cyclophosphamide; H & E: Hematoxylin and eosin; IMPC: Invasive micropapillary carcinoma; IS: Intensity score; LH-RH: Luteinizing hormone-releasing hormone; MRI: Magnetic resonance imaging; MUC-1: Mucin 1 cell surface associated; NCC-ST439: National Cancer Center-ST439; PgR: Progesterone receptor; PS: Proportion score; TS: Total score; UICC: Union for International Cancer Control; VEGF: Vascular endothelial growth factor.

## Competing interests

The authors declare that they have no competing interests associated with this study.

## Authors’ contributions

KT drafted the manuscript. YZ and SA carried out the immunohistochemical assay and pathological examination. MT participated in the sequence alignment. ES participated in the design of the study and performed the statistical analysis. ET, NY, KT, SA, SO, MM, and YM participated in its design and coordination and helped to draft the manuscript. All authors read and approved the final manuscript.
